# Supplementary material for: Probation as a targeted entry point for scaling up opioid agonist therapies in Moldova using a modified screening, brief intervention and referral to treatment strategy
Source: PLOS Glob Public Health. 2026 May 27;6(5):e0006317. doi: 10.1371/journal.pgph.0006317 (PMC13215523; doi:10.1371/journal.pgph.0006317)
Supplement: Attachment — Submitted filename: PLOS_Review.docx [file pgph.0006317.s001.docx]

The submitted manuscript addresses a relevant topic and an important opportunity to scale-up OAT programs in Moldova through the probation system. Also, this article reflects the results of one of two studies conducted using the same design in parallel, the second study was conducted in Ukraine. Both countries have similar historical backgrounds and, as a result, similar legal and prison system problems and characteristics of the HIV epidemic, which has been concentrated among people who inject drugs for decades. Therefore, conducting such studies and publishing articles based on their findings is crucial. However, despite the many systemic similarities between Ukraine and Moldova, there are also significant differences, particularly in the implementation of the probation system, which was established in Moldova much earlier than in Ukraine. I focus on research in Ukraine, specifically in prisons, but I am also familiar with the prison contexts of the surrounding countries. In particular, I know that until very recently, the main probation efforts in Moldova focused on penitentiary probation, which represented a series of measures taken by prison officers shortly before the release of people serving prison sentences. In Ukraine, on the contrary, significant efforts were directed at establishing probation as an alternative to imprisonment. The article does not explain what type of probation is being discussed or how it is organized at all, and individual phrases that do not constitute a general context only confuse understanding. As it stands now, the text itself is substantively lacking in contextual data, and more broadly–given that the two studies were conducted in two countries in parallel using the same protocol–in methodological consistency. For example, the Study setting subsection states that

The probation system combines 38 probation offices(41) to oversee persons deemed stable enough to be supervised in community settings. It is linked to social rehabilitation centers that supplement these services in three territorial regions: north, center and south. At the end of 2018, there were 11,970 people on probation(42). (147-151, p. 6)

Here it is necessary to clarify what these social rehabilitation centers are, since you mention them, and how they differ from prisons, and what civil status do the people who stay in them or systematically visit them have? And what is the connection between these 38 offices and the three social rehabilitation centers? And under what conditions can people get into probation, if it is an alternative to imprisonment (e.g. for minor offenses, first encounter with the criminal system, etc.).

Further in the Results section, in Table 1. Baseline participant characteristics, stratified by initiation of OAT (N = 119), one of the participant characteristics is Previously held in pre-trial detention center, but what does this characteristic tell us if we do not understand the conditions of probation entry and what type of probation we are talking about? In any case, it seems strange to ask only whether the participant has been Previously held in pre-trial detention center (SIZO) without asking whether he/she has been previously held in prison(colony), unless the mere possibility of being placed on probation excludes the possibility of having previous experience of imprisonment in a colony. In any case, this question should be clarified.

Finally, in the Discussion section, the authors point out that

probation is an extremely stressful period for individuals with low social support. This may suggest the need to create structural interventions that pay attention to what drives subcultural norms, including social, physical and economic levels(66) in order to address them before people in prison are moved to the probation period. (425-429, p. 22)

Due to the lack of information about the conditions of probation, the first statement looks too strong and in no way linked to the data discussed in the article. In the next sentence, it says that ‘in order to address them before people in prison are sentenced to the probation period’, which means that the authors were talking about penitentiary probation, i.e. prison-based probation? Why then do the authors go on to write that

While little is known about how dynamics brought on by criminal subculture persist outside of the prison setting and into the probation period, we can hypothesize that criminal subcultural influence is lower in probation than in prisons, but we do know that these norms continue into the community and may impact probation’ (429-432, p. 22).

Although here I would like to point out that this phrase once again confuses the understanding of how the probation system is implemented in Moldova, I would also like to ask why attitudes learned while serving prison time (*ponyatiya*) cannot be transferred into life after people are released? Does Moldova, as in other post-Soviet countries, have an effective system of social re-integration of former prisoners, which allows people not to return to similar communities outside the prison walls?

Although I have no questions about the reliability of the findings, I would like to draw the authors' attention to the internal contextualization of the study results, which opens up new possibilities for interpreting the data. In particular, this may help to better understand some of the issues that the authors raise in the discussion, such as the low motivation of participants to engage in OAT or the persistence of negative attitudes about OAT. For example, in the Results section, Table 1. Baseline participant characteristics, stratified by initiation of OAT (N = 119), among participant characteristics is the parameter ‘Registered at narcology addiction treatment center’. It is further mentioned in Table 6: Reasons for not initiating OAT among people who did not initiate OAT (N = 86). Out of 86 people who did not initiate OAT, only six indicated that they ‘Did not want to get registered’. If we go back to Table 1, to the ‘Registered at narcology addiction treatment center’ characteristic, we can notice that out of 86 people who did not initiate OAT, only 18 responded that they were not ‘Registered at narcology addiction treatment center’. Therefore, considering ‘'Did not want to get registered’ as a reason for not initiating OAT can only be relevant for the 18 people who were not registered before. Whereas 6 out of 18 is a very different proportion and therefore a very different position for this reason in the overall hierarchy of reasons for not initiating OAT treatment. I would recommend the authors to pay special attention to the topic of mandatory registration of people with OUD to be eligible for OAT. A number of studies in Ukraine have addressed this issue, where the emergence of private OAT programs, which do not always require confirmation of OUD diagnosis, automatically removes the problem of mandatory registration if people have the money to pay for the services of a private OAT program.

I would like to draw the attention of the authors to the use of the concept ‘criminolegal’, in particular in the case of ‘criminolegal settings’ in abstract and several times in the text and ‘From a criminolegal perspective’ in Introduction, p. 3, 63. If there is some sense in using this particular concept, which most resembles the calque from Russian (*ugolovno-pravovoy*), which may have diffused into the normative documents of other Soviet countries during the Soviet era, then it is worth explaining its use. If not, it is worth replacing it with a more widely accepted one.

One last minor point. The Results section begins with a subsection on Participant disposition, stating that ‘Forty-sever (39.5%) overdosed in the last 6 months’. The authors do not revisit this result again, then why is it important to mention here? While it also seems important to me, I would like to see the authors' comments in the discussion.

I would like to end by saying that, as I wrote at the beginning, the publication of this article is both important and needed, and I hope that my comments will help to make this text even better.
