# Supplementary material for: Probation as a targeted entry point for scaling up opioid agonist therapies in Moldova using a modified screening, brief intervention and referral to treatment strategy
Source: PLOS Glob Public Health. 2026 May 27;6(5):e0006317. doi: 10.1371/journal.pgph.0006317 (PMC13215523; doi:10.1371/journal.pgph.0006317)
Supplement: Attachment — Submitted filename: Response to Reviewer_PGPH_Moldova_Final (1).docx [file pgph.0006317.s002.docx]

September 1^st^, 2025

Professors Catherine Kyobutungi and Madhukar Pai

Editor, *PLOS Global Public Health*

Dear Drs. Kyobutungi and Pai,

We would like to thank the reviewer for their thoughtful review of our manuscript entitled “*Probation as a targeted entry point for scaling up opioid agonist therapies in Moldova using a modified screening, brief intervention and referral to treatment strategy”* (**PGPH-D-24-02730**). Below, we provide point-by-point responses to each of their comments and suggestions, including italicized excerpts from the revised text and the line numbers where we have implemented the requested changes.

**Comment**: As it stands now, the text itself is substantively lacking in contextual data, and more broadly–given that the two studies were conducted in two countries in parallel using the same protocol–in methodological consistency. For example, the Study setting subsection states that

The probation system combines 38 probation offices(41) to oversee persons deemed stable enough to be supervised in community settings. It is linked to social rehabilitation centers that supplement these services in three territorial regions: north, center and south. At the end of 2018, there were 11,970 people on probation (42). (147-151, p. 6)

Here it is necessary to clarify what these social rehabilitation centers are, since you mention them, and how they differ from prisons, and what civil status do the people who stay in them or systematically visit them have? And what is the connection between these 38 offices and the three social rehabilitation centers? And under what conditions can people get into probation, if it is an alternative to imprisonment (e.g. for minor offenses, first encounter with the criminal system, etc.).

**Response:** We thank the reviewer for the opportunity to clarify. We have added the following to add more detail and clarification:

Moldova’s probation service operates under Law No. 8 since 14 February 2008, **which outlines the organization, mandates, and scope of probation services, including their responsibilities in rehabilitation and oversight** **(55)**. These services are independent of prisons and all are delivered exclusively in community settings, enabling probation officers to engage flexibly with family networks, community health and social services, and employment opportunities. The central National Probation Inspectorate (NPI) oversees 38 territorial probation offices, which are organized into three regional inspectorates in the North, Centre, and South regions. Among these offices, only three include “centers for social rehabilitation.” These centers are strictly administrative and serve as daytime locations for delivering probation-related rehabilitation programs, not as detention facilities.

Individuals under probation may include: persons undergoing pre‑trial psychosocial evaluations; those conditionally or post‑released from criminal sentences; individuals serving unpaid community service; and individuals subject to protective or family-related court orders. Placement on probation is determined by the court, based on the case facts and aligned with the Criminal Code’s provisions. For example, unpaid community service may be imposed as a standalone community sanction or as part of a suspended sentence.

**Comment**: Further in the Results section, in Table 1. Baseline participant characteristics, stratified by initiation of OAT (N = 119), one of the participant characteristics is Previously held in pre-trial detention center, but what does this characteristic tell us if we do not understand the conditions of probation entry and what type of probation we are talking about? In any case, it seems strange to ask only whether the participant has been Previously held in pre-trial detention center (SIZO) without asking whether he/she has been previously held in prison(colony), unless the mere possibility of being placed on probation excludes the possibility of having previous experience of imprisonment in a colony. In any case, this question should be clarified.

**Response:** Thank you for the opportunity to clarify. Participants could answer “yes” or “no” to whether they had been previously held in pre-trial detention, also known as SIZO. We overlooked including past experiences with incarceration in a prison (colony) and have added it to Table 1.

**Comment**: Finally, in the Discussion section, the authors point out that

probation is an extremely stressful period for individuals with low social support. This may suggest the need to create structural interventions that pay attention to what drives subcultural norms, including social, physical and economic levels(66) in order to address them before people in prison are moved to the probation period. (425-429, p. 22)

Due to the lack of information about the conditions of probation, the first statement looks too strong and in no way linked to the data discussed in the article. In the next sentence, it says that ‘in order to address them before people in prison are sentenced to the probation period’, which means that the authors were talking about penitentiary probation, i.e. prison-based probation? Why then do the authors go on to write that

While little is known about how dynamics brought on by criminal subculture persist outside of the prison setting and into the probation period, we can hypothesize that criminal subcultural influence is lower in probation than in prisons, but we do know that these norms continue into the community and may impact probation’ (429-432, p. 22).

Although here I would like to point out that this phrase once again confuses the understanding of how the probation system is implemented in Moldova, I would also like to ask why attitudes learned while serving prison time (*ponyatiya*) cannot be transferred into life after people are released? Does Moldova, as in other post-Soviet countries, have an effective system of social re-integration of former prisoners, which allows people not to return to similar communities outside the prison walls?

**Response:** We agree and have provided more subtext throughout and revised our discussion accordingly.

**Comment**: I would recommend the authors to pay special attention to the topic of mandatory registration of people with OUD to be eligible for OAT. A number of studies in Ukraine have addressed this issue, where the emergence of private OAT programs, which do not always require confirmation of OUD diagnosis, automatically removes the problem of mandatory registration if people have the money to pay for the services of a private OAT program.

**Response:** We now include a section in the country context for the role of the national registry as well as include this as a limitation for why more people did not enroll on MOUD – along with key citations.

Lines 309-313: To enroll on MOUD, it is required that a person with OUD be diagnosed by an addiction treatment specialist and placed on a national registry. Once on this registry, and while on MOUD, a person’s personal liberties are restricted as they are ineligible for a driver’s license and restricted from some types of employment (e.g., operating heavy equipment). This national registry, much like throughout all of EECA, is a major impediment to MOUD enrollment(35,37,38,59–61).

**Comment:** I would like to draw the attention of the authors to the use of the concept ‘criminolegal’, in particular in the case of ‘criminolegal settings’ in abstract and several times in the text and ‘From a criminolegal perspective’ in Introduction, p. 3, 63. If there is some sense in using this particular concept, which most resembles the calque from Russian (*ugolovno-pravovoy*), which may have diffused into the normative documents of other Soviet countries during the Soviet era, then it is worth explaining its use. If not, it is worth replacing it with a more widely accepted one.

**Response:** We use the criminal legal system to avoid terminology like criminal justice system, which implies that the laws are just. This is in accordance with a growing body of literature. Here are a few examples to justify the use of this terminology. If the editors prefer us to use a different term, we can make this change, but the current one is the one most recently recommended.

A. Benjamin Levin, After the Criminal Justice System, 98 Wash. L. Rev. 899 (2023).
B. Cao M, Esposito M, Lee H. The U.S. Criminal Legal System and Population Health. Curr Epidemiol Rep 2025; 12.

**Comment:** One last minor point. The Results section begins with a subsection on Participant disposition, stating that ‘Forty-seven (39.5%) overdosed in the last 6 months’. The authors do not revisit this result again, then why is it important to mention here? While it also seems important to me, I would like to see the authors' comments in the discussion.

**Response:** We have added the following in the Discussion to address this comment,

Lines 543 – 554: “Notably, nearly 40% of participants reported experiencing an overdose within the past six months, a critical indicator of elevated risk for morbidity and mortality within this supervised population. Research indicates that individuals under probation face overdose rates **up to 15 times higher** than the general population, and overdose fatalities remain strikingly high during periods of community supervision.(11,12) This alarming prevalence underscores the urgent need for enhanced surveillance of opioid use disorder and rapid linkage to **OAT** like methadone, buprenorphine,(69) which are proven to substantially reduce overdose mortality(70) and help reduce the transmission of blood-borne infections like HIV and HCV(2–4). This finding further highlights an important opportunity for probation officers to play a pivotal role in harm reduction efforts through proactive screening for substance use, facilitating access to OAT, and distributing naloxone to protect both individuals and the wider community”

Sincerely,

Matthew Ponticiello

MD-PhD Candidate

Yale School of Medicine and Public Health
