# Supplementary material for: Probation as a targeted entry point for scaling up opioid agonist therapies in Moldova using a modified screening, brief intervention and referral to treatment strategy
Source: PLOS Glob Public Health. 2026 May 27;6(5):e0006317. doi: 10.1371/journal.pgph.0006317 (PMC13215523; doi:10.1371/journal.pgph.0006317)
Supplement: Attachment — Submitted filename: R2R Revision 2_11_14_25_FINAL.docx [file pgph.0006317.s003.docx]

November 14^th^, 2025

Professor Kyobutungi and Pai

Editors-in-Chief, *PLOS Global Public Health*

Dear Drs. Kyobutungi and Pai,

We would like to thank the reviewer for their thoughtful review of our manuscript entitled “*Probation as a targeted entry point for scaling up opioid agonist therapies in Moldova using a modified screening, brief intervention and referral to treatment strategy”* (**PGPH-D-24-02730**). Below, we provide point-by-point responses to each of their comments and suggestions.

**ABSTRACT**

**Comment 1:**
In Methods or Results, would be helpful to very briefly detail the ‘MOUD interest score’ – for example, is this a validated instrument or 1 question and what is the possible score range and directionality?

**Response:** We have added the following to Methods, “MOUD interest was assessed using a single-item screening instrument where higher scores were associated with greater interest in starting MOUD.”

**Comment 2:**
Just a note that any changes below should be integrated into the Abstract, where appropriate.

**Response:** Noted

**INTRODUCTION**

**Comment 3:**
Would add that another important individual and public health benefit of MOUD is decreased risk of overdose.

**Response:** We have added, “…as well as decreased risk of overdose.”

**Comment 4:**
Suggest adding a bit more information on the typical MOUD programs available in Moldova – methadone predominantly? Free-of-charge from the public sector? In hospitals/clinics or community-based locations?

**Response:** Both methadone and buprenorphine are available in Moldova, including within penitentiary settings. There are currently 13 MOUD sites in prisons and 9 community-based sites, mostly integrated within narcological (addiction treatment) services, treatment is offered free of charge through the public health sector. The procurement of medications is financed from the state budget, while the psychosocial support component remains largely donor-funded and implemented by NGOs.

**Comment 5:**
For those unfamiliar with the legal system, would be good to clarify whether those going into probation are coming out from prison or straight from sentencing – or a mix. Or maybe that is what ‘post-released from criminal sentences’ means – suggest revision to clarify.

**Response:** People with HIV and opioid use disorder (OUD) are concentrated in criminal legal settings. Consequently, decarceration efforts in Moldova have shifted many people from prisons to community supervision under probation. In Moldova, probation applies both to individuals released from prison before completing their sentence and to those sentenced directly to non-custodial supervision. We have clarified this in Lines 185 – 186.

**Comment 6:**
The authors mention that one of primary goals of the study was to pilot test the feasibility of targeting probation as an opportunity to encourage MOUD, but it isn’t clear after reading the paper how ‘feasibility’ is defined or assessed. Suggest the authors more clearly define what their aims are for this paper.

**Response:** We have left the sentence as is to reflect this was indeed a feasibility study although implementation outcomes, specifically, were not collected.

**METHODS**

**Comment 7:**
Much of the extensive background on probation in Moldova seems better suited for the Introduction than Methods and there is likely more detail than required (e.g., probably don’t need to know the Law No. that governs probation, the required education for probation officers) – but defer to editor on these.

**Response:** Thank you for this note. A previous reviewer request more in-depth information on these topics, hence their inclusion. Thus, we have opted to maintain a section on context to focus on this aspect of implementing the program in probation (Lines 186 – 199).

**Comment 8:**
This sentence is not clear as currently written: “To avoid potential conflict between officer and client, the research team was allowed to be present in the office where the officer typically meets with clients every 2-4 weeks.” How does this avoid conflict between the client and officer?

**Response:** “To avoid potential conflicts between the officer’s supervisory role and the research procedures, the research team conducted study activities in the same office where the officer typically meets clients every 2–4 weeks, but all research procedures were carried out privately by the research team, independent of probation staff.” (Lines 202 – 206). This revision clarifies that the arrangement was intended to reduce role conflict and ensure confidentiality during study activities.

**Comment 9:**
Related to the daily caseloads of probation officers – these seem really high but maybe the interpretation of ‘daily’ could be clarified as it could be interpreted as the number of people the officers see/interact with each day.

**Response:** In the manuscript, we mentioned the offices rather than the officers, because it is challenging to provide an accurate individual case load as some clients may have more challenging needs and require more time, while more stable probationers may be seen quickly. We therefore clarify that there are 272 probation officers in 26 probation offices, but that capacity and schedules differ for each officer.

**Comment** **10:**
Participants – Would add date range in which clients were approached (assuming enrollment was not the full study period [01/11/2019 to 30/04/2023]?)

**Response:** The last participant was enrolled six months before the end of the study period, which we have now clarified, as each participant was observed for a full 6 months.

**Comment 11:**
Participants – If space allows – would suggest the authors include the full text of the ‘single item screening question’ for opioid use. And, is this just for opioid use or opioid use disorder?

**Response:** The question includes opioid use only **-** the frequency of nonmedical use of illicit opioids over the past year.

**Comment 12:**
Participants – The authors detail the confirmation of opioid use disorder in the next section but would very briefly mention this in the ‘Participants’ section. Maybe within the inclusion criteria – e.g., ‘OUD diagnosis using the brief Rapid Opioid Dependence Screen’ – if this interpretation of screening and inclusion is correct.

**Response:** We have added clarification in the Participants section to indicate that OUD diagnosis was confirmed using the brief Rapid Opioid Dependence Screen following a positive single-item screen for opioid use.

**Comment 13:**
Suggest removing ‘Study procedures are similar as those described in Ukraine(40) as this was part of a two-site study’, as this has already been mentioned.

**Response:** This has been removed accordingly.

**Comment 14:**
‘Both methadone and buprenorphine were offered as treatment’ (line 291-292) – this seems oddly placed and sounds as if the research assistants were offering treatment in the probation office. Suggest that authors revise.

**Response:** Thank you for this note. We have moved up the phrase to a place where it is clearer that we are expressing methadone and buprenorphine are offered in general as part of the study and not that RAs are offering the treatment.

**Comment 15:**
‘In this study, the only MOUD available are opioid agonist therapies (OAT) including methadone or buprenorphine… Moldova’s Ministry of Health provides MOUD for free in prisons and within the community, including methadone and buprenorphine…’ – these sentences are oddly placed (better in Introduction, probably) and confusing as written. Did the study provide MOUD or are the authors referring to the publicly available MOUD?

**Response:** The authors are referring to publicly available MOUD. We have made this clearer; however, kept the sentences in Moldovan context(Lines 194 – 196).

**Comment 16**
Were those currently uninterested in MOUD at least given referral information? If not, why?

**Response**: We have added the following, “Individuals who were not interested in initiating MOUD at the time of screening were provided with referral information for harm reduction and other relevant health services available through NGOs and medical units. This included details on needle and syringe programs, HIV testing and care, psychosocial support, and MOUD services for those who may wish to engage in treatment later. A comprehensive directory of harm reduction centers operating in Moldova was developed and shared with probationers. The document outlines health risks associated with opioid dependence and provides guidance on accessing community-based services for drug treatment, social support, HIV and hepatitis C testing, and other health services. The information package also includes a list of all community-level health and harm reduction services available nationwide” (Lines 286 – 295).

**Comment 17:**

‘Participants could enroll in MOUD up to 6 months after their brief intervention’ (lines 304-305). It is unclear why clients would have MOUD access restricted at any point. Maybe the authors meant that they assessed MOUD initiation up to 6 months after some time point (specific time point being X)? Relatedly, for MOUD retention, participants were followed for 6 months following what time point (study enrollment or MOUD initiation?). Suggest authors revise for clarity.

**Response:** There are no limitations in MOUD initiation, and the text was adapted to, “There were no limitations on MOUD initiation and for anyone who initiated MOUD, they were followed for 6 months from baseline (and the SBIRT) to assess retention in MOUD.”

**Comment 18:**
If space allows, it would be helpful to understand what exact data was extracted from the MOUD clinical sites (medication prescribed, refills dates, etc.) and how study staff were able to link study participants to patient records (e.g., unique ID, name).

**Response:** Staff were able to link study participants to patient records through a unique registration identifier in MOUD provided to the research team by the study participant (Lines 308 – 309).

**Comment 19:**
‘but not beyond that’ (lines 308-309) is not needed and suggest removing.

**Response:** Removed.

**Comment 20:**
More details on the lab study procedures is needed – where was the rapid testing and counseling completed? The probation office? Was the HCV testing antibody only or also RNA? What was the syphilis testing exactly – RPPR and/or TPHA?

**Response:** NGO-based harm reduction service providers conducted rapid, point-of-care testing and counseling or assisted the self-testing. Probation staff facilitated referrals, while testing was performed either on-site at the probation office during outreach sessions or at nearby community harm reduction centers operated by NGOs. HIV and HCV screening using rapid antibody tests. Individuals who tested HCV antibody–positive were further referred to local clinics for confirmatory HCV RNA testing through the national public health laboratory network. Syphilis testing was performed using rapid plasma reagent (RPR) screening test. (Lines 339 – 344)

**Comment 21:**
There is an insufficient amount of information provided on what exactly the intervention entailed besides that it was motivational interviewing. This section needs considerable attention and revision so that readers can put findings in context. Also, for the sake of clarity, suggest that the details on the intervention (i.e., motivational interviewing) be its own section separate from data collection procedures.

**Response:**

**Brief Intervention (BI)**

The time of the brief intervention lasted up to 30 minutes, allowing participants to ask questions. The participants met one to one with a trained interviewer to get information under the “brief intervention” component of the research. The brief intervention has four objectives:

1. Identify the level of interest in MOUD, to focus on elements critical in motivational interviewing (Bromberg et al., 2023; Fisher et al., 2011).
2. To address information deficits related to the dangers of psychoactive substance use by informing them about the potential hazards and negative health effects with ongoing use and the benefits of MOUD.
3. To motivate the participants to reduce substance use and related risk behaviors, including the use of MOUD and syringe services programs.
4. To support behavioral skills to access risk reduction services.

After the brief intervention, the researcher re-assessed the interest of the participant to start the MOUD. If the participant accepted MOUD, each participant was interviewed at baseline (Day A) and followed up at 1, 3, and 6 months by the same interviewer, who tracks timelines in Tracking Form 1. After Day A, interviewers maintain contact with participants and probation officers to schedule interviews within the window period (two weeks before and after the target date). Missed or delayed interviews and reasons are documented. At each follow-up, interviewers conducted a REDCap interview on tablet/computer and verify OAT enrolment or assess interest in OAT. All data are linked to unique participant codes to ensure confidentiality. Interviewers receive monthly online training and coordinate with probation staff and the research team to update participant status, including any detention or deregistration. (Lines 392 – 729) [There is a glitch in the document disrupting appropriate line numbering].

**Comment 22:**
The scale mentioned for difficulty in accessing MOUD and importance of starting MOUD seems not to match – ‘0 indicated no interest at all and 10 indicated the highest level of interest possible.’ This appears to just be the scale for interest. Suggest authors revise.

**Response:** We have revised the description of these scales to clarify that all three measures—interest in MOUD, perceived difficulty in accessing MOUD, and perceived importance of initiating MOUD—where each rated from 0 to 10, with higher scores indicating greater levels of the construct assessed (e.g., higher importance, greater perceived difficulty, or higher interest). (Lines 351 – 354)

**Comment 23:**
For what is assumed to be the primary outcome of interest – the ‘MOUD interest score’ – the authors mention that it was measured ‘before and after the brief intervention’ – does ‘after’ mean immediately after the baseline intervention session (same day as the baseline visit) or after the second session which was 30 days after the initial intervention. This timeframe is very unclear as written. Additionally, has this ‘score’ been validated?

**Response: Interest in methadone was not the primary outcome – enrollment on MOUD was. For clarification, interest in** MOUD was measured before and after the brief intervention. The level of interest in MOUD, based on motivational interviewing techniques, is used as a guide to engage the client in discussion. This measure has been previously used in other MI studies.^1, 2^

1.	Fisher JD, Amico KR, Fisher WA, Cornman DH, Shuper PA, Trayling C, Redding C, Barta W, Lemieux AF, Altice FL, Dieckhaus K, Friedland G, LifeWindows T. Computer-based intervention in HIV clinical care setting improves antiretroviral adherence: the LifeWindows Project. AIDS Behav. 2011;15(8):1635-1646. PMID: 21452051.

2.	Bromberg DJ, Galvez de Leon SJ, Litz T, Azbel L, Liberman AR, Polonsky M, Dvoriak S, Saichuk N, Taxman F, Altice FL. Aligning public health and public safety: Probation as a touchpoint to identify and link patients with opioid use disorder to opioid agonist treatment. PLOS Glob Public Health. 2023;3(11):e0002349. PMID: 37910486; PMCID: PMC10619786.

**Comment 24:**
Line 323 – the word ‘standardized’ is repeated.

**Response:** We removed the word standardized.

**Comment 25:**
It is unclear what the alpha’s are in lines 325-326. If these are Cronbach’s alphas related to the consistency of the scale in this study, suggest this be presented in Results (with appropriate details in the Methods).

**Response:** We measured Cronbach’s alpha and have now included this.

**Comment 26:**
First, this sentence: ‘A receiver operator curve (ROC)… in initiating MOUD’ (lines 333-334) should be below in the Stats Analysis section. But, more importantly, this approach is missing critical details such as what is the outcome used, as the ROC is classifying a dichotomous state/outcome (positive/negative for something), and what was the purpose of this ROC (what purpose does it play in the research question).

**Response:** We have moved the ROC analysis to the Statistical Analysis section and expanded the description to specify that the ROC was used to determine the optimal cutoff point on the 0–10 MOUD interest scale for predicting subsequent MOUD initiation. The area under the curve (AUC = 0.8554) identified a score of 8 as the most discriminative threshold and this was used in subsequent analyses. (Lines 388 – 390)

**Comment 27:**
Then, the next sentence ‘The AUC was 0.8554… Appendix).’ should be in the Results.

**Response:** We have moved this sentence to the Results section as suggested.

**Comment 28:**
Suggest the following changes for the Stats Analysis section:

1. Clearly state and define primary and secondary outcomes,
2. Link each analysis to a scientific question,
3. Mention significance level and stats software.

**Response:** We thank the reviewer for their suggestion but have left the stats section as is.

**Comment 29:**
Suggest authors describe how they picked predictive variables of MOUD initiation and what they were.

**Response:** We added text specifying that variables were selected based on theoretical relevance and prior literature on predictors of MOUD uptake, including sociodemographic characteristics, HIV and HCV status, prior treatment experience, and perceived importance of MOUD (Lines 384 – 387).

**RESULTS**

**Comment 30:**
‘Forty-seven (39.5%) overdosed in the last 6 months.’ (lines 357-358) – it is unclear why this sentence is here as it seems better in ‘Participant characteristics.’

**Response:** We have moved the line regarding overdose to participant characteristics.

**Comment 31:**
‘Those who started MOUD were significantly more likely to be older, women, not have children, HIV, HCV or syphilis, relative to those who did not start MOUD.’ – this seems off relative to what is presented in Table 1. Suggest this sentence be re-written for clarity.

**Response:** We have revised this sentence to accurately reflect the findings presented in Table 1. It now reads:
“Those who started MOUD were significantly more likely to be older, female, have HIV or HCV infection, and to rate MOUD as an important treatment option compared with those who did not initiate MOUD.” (Lines 824 – 826)

**Comment 32:**
‘Using the cutoff of >=8’ – 8 what exactly? It seems the authors dichotomized the MOUD interest score maybe and analyzed in this manner but there are no details of this in the Methods.

**Response:** In motivational interviewing, a cutoff of 8 is indicative of high levels (1,2). We have now clarified in the Methods that the value of 8 referred to the cutoff score on the 0–10 MOUD interest scale derived from the receiver operator curve (ROC) analysis. This threshold distinguished participants highly interested in initiating MOUD and was used in subsequent analyses to predict MOUD initiation.

1.	Fisher JD, Amico KR, Fisher WA, Cornman DH, Shuper PA, Trayling C, Redding C, Barta W, Lemieux AF, Altice FL, Dieckhaus K, Friedland G, LifeWindows T. Computer-based intervention in HIV clinical care setting improves antiretroviral adherence: the LifeWindows Project. AIDS Behav. 2011;15(8):1635-1646. PMID: 21452051.

2.	Bromberg DJ, Galvez de Leon SJ, Litz T, Azbel L, Liberman AR, Polonsky M, Dvoriak S, Saichuk N, Taxman F, Altice FL. Aligning public health and public safety: Probation as a touchpoint to identify and link patients with opioid use disorder to opioid agonist treatment. PLOS Glob Public Health. 2023;3(11):e0002349. PMID: 37910486; PMCID: PMC10619786.

**Comment 33:**
‘The mean interest in MOUD score increased…’ – the analysis section does not mention the score being analyzed as a mean – rather the Wilcoxon signed-rank test was mentioned. Clarify in Methods, Results, and Tables.

**Response:** We revised the text to indicate that mean values were presented descriptively for interpretability, while statistical significance for paired differences was determined using the Wilcoxon signed-rank test. This clarification has been applied consistently in the Methods, Results, and table legends. (Lines 396 – 398)

**Comment 34:**
For Tables 4 and 5 – the authors only refer to the tables without detailing or summarizing the results. Either these results should be presented in the text or excluded.

**Response:** In line with most journals, data from tables should not be detailed line by line. Instead, we have provide a summary of the highlights. If the editor would prefer a different approach, we will defer to them – the other reviewers did not advocate for more details in the text.

**Comment 35:**
Change ‘multivariate’ to ‘multivariable’ analysis. Additionally, mention all included covariates in the logistic regression.

**Response:** We changed “multivariate” to “multivariable” throughout the text. We also specified the covariates included in the logistic regression model: age, sex, HIV status, HCV status, prior MOUD attempt, registration in the narcology registry, and post-intervention importance score for MOUD (Lines 747 – 749).

**Comment 36:**
Why are details on OAT initiation bulleted? Suggest authors integrate this into the text.

**Response:** We have integrated these details into the Results text for smoother narrative flow. The information on OAT initiation and retention is now summarized in paragraph form rather than as bullet points.

**TABLES/FIGURES**

**Comment 37:**
Most table titles are too vague – specify who the participants are.

**Response:** We revised all some tabes titles to specify that the data refer to “participants on probation with opioid use disorder” to improve clarity and contextual understanding (Tables 1-4).

**Comment 38:**
Table 1 – Dichotomous variables don’t need both yes and no rows.

**Response:** We reformatted Table 1 by removing redundant “yes/no” rows for dichotomous variables to enhance readability.

**Comment 39:**
Table 1 – ‘Previously attempted to receive OAT’ is vague; suggest clarifying whether it includes those who tried but never started OAT.

**Response:** We clarified that “previously attempted to receive OAT” includes participants who had previously sought treatment but did not initiate or complete OAT.

**Comment 40:**
Table 1 – Was treatment status collected from those with HIV, HCV, syphilis, depression? If so, add to table.

**Response:** Those data were not collected.

**Comment 41:**
Table 1 – Details on some scales/instruments in this table are missing from the Methods (DAST-10, social support scale).

**Response:** We added a description of the DAST-10 and social support scale instruments in the Methods section, including scoring procedures and interpretation, to ensure alignment with the variables presented in Table 1.

**Comment 42:**
Figure 2 – ‘Interested in OAT-Pre/Post’ appears twice and the ‘brief intervention’ line seems misplaced. Suggest revision.

**Response:** We thank the reviewer for their notes. The previous reviewer provided no additional comments on the figures so we will maintain them as is unless the editors request otherwise.

**DISCUSSION**

**Comment 43:**
Much of the detailed discussion on how SBIRT could be implemented in probation settings seems out of scope. Suggest focusing on study objectives, findings, and implications.

**Response:** We condensed the discussion of SBIRT implementation strategies to focus primarily on our study’s key findings and their implications for probation settings in Moldova. We do, however, speculate on some of the challenges in this setting, which are important for future implementers. We retained a concise paragraph on implementation opportunities to contextualize scalability without overextending beyond the study’s scope.

**Comment 44:**
‘brief interventions have demonstrated little benefit related to drug use’ – evidence is mixed; revise phrasing per recommended citation.

**Response:** We revised the phrasing to reflect the mixed evidence base, now stating: “Although evidence for the effectiveness of brief interventions in reducing drug use is mixed, they do appear to have benefit within SBIRT protocols for alcohol and OUD. In this study, the findings align with studies showing modest improvements in motivation but limited effects on behavioral outcomes” (Lines 993 – 996). Nonetheless, the brief intervention in some SBIRT involving alcohol and direct access to MOUD have, however, been effective.

**Comment 45:**
‘In contrast to both these studies…’ – clarify which studies are being referred to; these cannot be the present and Ukraine studies.

**Response:** We clarified that the comparison refers to the Moldova and Ukraine studies conducted using the same modified SBIRT methodology. The sentence now explicitly identifies these studies by country for clarity.

**Comment 46:**
Add limitations of pre/post design versus RCT with control group.

**Response:** We added to the Limitations section that the pre/post design without a control group limits causal inference regarding intervention effects on MOUD interest and initiation compared with randomized or controlled designs (Lines 1080 – 1082).

**Comment 47:**
Clarify statement about national registry influence on MOUD initiation; current phrasing unclear.

**Response:** We revised the statement to clarify that Moldova’s national registry for people with OUD—which imposes restrictions on employment and driving—acts as a structural deterrent to MOUD initiation and may partially explain the modest uptake observed despite expressed interest (Lines 1078 – 1079).

**Comment 48:**
The findings do not support this sentence: ‘…this modified SBIRT strategy deployed in probation by trained interventionists was effective at improving the uptake of MOUD.’ Revise to reflect actual findings (change in interest, not uptake).

**Response:** We revised the sentence to more accurately reflect our results:
“This modified SBIRT strategy deployed in probation by trained interventionists was effective in identifying individuals with OUD and increasing their interest in MOUD. The intervention also resulted in a substantial increase in the number of people with OUD initiating MOUD relative to before the intervention” (Line 1088 - 1091).

Please do not hesitate to reach out should you have any additional questions.

Best,

Matthew Ponticiello
