# Supplementary material for: Probation as a targeted entry point for scaling up opioid agonist therapies in Moldova using a modified screening, brief intervention and referral to treatment strategy
Source: PLOS Glob Public Health. 2026 May 27;6(5):e0006317. doi: 10.1371/journal.pgph.0006317 (PMC13215523; doi:10.1371/journal.pgph.0006317)
Supplement: Attachment — Submitted filename: R2R Revision 3_FINAL.docx [file pgph.0006317.s004.docx]

December 11^th^, 2025

Professor Kyobutungi and Pai

Editors-in-Chief, *PLOS Global Public Health*

Dear Drs. Kyobutungi and Pai,

We would like to thank both reviewers for their thoughtful review of our manuscript entitled “*Probation as a targeted entry point for scaling up opioid agonist therapies in Moldova using a modified screening, brief intervention and referral to treatment strategy”* (**PGPH-D-24-02730**). Reviewer 1 believed that all the criticisms had been addressed. For the other review, we provide point-by-point responses to each of their comments and suggestions.

**ABSTRACT**

Comment 6:
**Initial comment:** The authors mention that one of primary goals of the study was to pilot test the feasibility of targeting probation as an opportunity to encourage MOUD, but it isn’t clear after reading the paper how ‘feasibility’ is defined or assessed. Suggest the authors more clearly define what their aims are for this paper.
**Authors’ response**: We have left the sentence as is to reflect this was indeed a feasibility study although implementation outcomes, specifically, were not collected.
**Reviewer response**: I will continue to push on this point. As the paper is laid out now, noting that a primary goal of the research is assessing feasibility is misleading as there are no results currently that address feasibility. What does feasibility mean in the context of THIS work?

**Authors’ response:** We thank the reviewer for pushing us to clarify how feasibility is defined and assessed in this study. We agree that our original wording implied a broader implementation feasibility than was evaluated. We have revised the manuscript to explicitly define feasibility in the context of this work as the feasibility of probation as a public health opportunity and the feasibility of core processes (screening, brief intervention delivery by external staff, and linkage to MOUD), rather than feasibility of implementation by probation personnel. We now clarify that implementation outcomes such as adoption, fidelity, and sustainability were not assessed and are beyond the scope of this study. We have also revised the Introduction, Methods, and Discussion to align feasibility claims with the outcomes measured and included this as a limitation.

Comment 26:
**Initial comment:** First, this sentence: ‘A receiver operator curve (ROC)... in initiating MOUD’ (lines 333-334) should be below in the Stats Analysis section. But, more importantly, this approach is missing critical details such as what is the outcome used, as the ROC is classifying a dichotomous state/outcome (positive/negative for something), and what was the purpose of this ROC (what purpose does it play in the research question).
**Authors’** **Response**: We have moved the ROC analysis to the Statistical Analysis section and expanded the description to specify that the ROC was used to determine the optimal cutoff point on the 0–10 MOUD interest scale for predicting subsequent MOUD initiation. The area under the curve (AUC = 0.8554) identified a score of 8 as the most discriminative threshold and this was used in subsequent analyses. (Lines 388 – 390)
**Reviewer response**: The revised text in the manuscript remains unclear; the above explanation which includes what the ‘gold standard’ is (i.e., documented MOUD initiation among enrolled study participants) is better and should be presented as such in the manuscript. Since the interest score was collected twice from each participant, were all of these data points used in the AUROC or just the first? Suggest detailing this process a bit more so readers can follow what was done.

**Authors’ response:** We thank the reviewer for this helpful clarification request. We have revised the Statistical Analysis section to explicitly describe the purpose, outcome, and analytic unit of the ROC analysis. The ROC was used to evaluate the ability of baseline self-reported MOUD interest (0–10 scale) to predict subsequent documented MOUD initiation (yes/no), which served as the gold-standard outcome. We now clarify that only the baseline interest score was included in the ROC analysis to preserve temporal ordering and avoid correlated observations from repeated measures. We also describe how the optimal cutoff (≥8) was selected using the Youden index and subsequently applied in downstream analyses. These revisions are intended to make the analytic process transparent and reproducible.

Comment 28:
**Initial Comment:** Suggest the following changes for the Stats Analysis section:
1. Clearly state and define primary and secondary outcomes,
2. Link each analysis to a scientific question,
3. Mention significance level and stats software.
**Authors’ Response**: We thank the reviewer for their suggestion but have left the stats section as is.
**Reviewer response:** I will continue to push on this point as well. The section is difficult to follow and should be revised for clarity.

**Authors’ response:** We thank the reviewer for this constructive feedback and agree that the Statistical Analysis section required clearer organization. We have substantially revised this section to (1) explicitly define primary and secondary outcomes, (2) link each analytic approach to its corresponding scientific question, and (3) specify the statistical significance threshold and software used. These revisions were made to improve clarity and transparency for readers.

Comment 33:
**Initial Comment:** ‘The mean interest in MOUD score increased...’ – the analysis section does not mention the score being analyzed as a mean – rather the Wilcoxon signed-rank test was mentioned. Clarify in Methods, Results, and Tables.
**Authors’ Response**: We revised the text to indicate that mean values were presented descriptively for interpretability, while statistical significance for paired differences was determined using the Wilcoxon signed-rank test. This clarification has been applied consistently in the Methods, Results, and table legends. (Lines 396 – 398).
**Reviewer response**: This approach – presenting a summary statistic that is not used in the statistical hypothesis test – is confusing and unnecessary. Conclusions such as this in the Discussion is not accurate: “Mean interest scores in starting MOUD increased significantly after the brief intervention.” Presentation of the median would be appropriate. Suggest revision.

**Authors’ response:** We thank the reviewer for this important clarification. We agree that presenting mean values alongside a nonparametric test was confusing. We have revised the manuscript to consistently present MOUD interest scores using medians and interquartile ranges and have updated the Methods, Results, Tables, and Discussion accordingly. All inference language now aligns with the Wilcoxon signed-rank test.

Comment 42:
**Initial Comment:** Figure 2 – ‘Interested in OAT-Pre/Post’ appears twice and the ‘brief intervention’ line seems misplaced. Suggest revision.
**Authors’ Response**: We thank the reviewer for their notes. The previous reviewer provided no additional comments on the figures so we will maintain them as is unless the editors request otherwise.
**Reviewer response**: This is an excellent example of why more than 1 (or 2) reviewers is so helpful to the process! It would behoove the authors to take some time to review the figure for accuracy (i.e., why would the brief intervention come AFTER the ‘post OAT interest assessment’?) and fix before publication.

**Authors’ response:** We thank the reviewer for this careful review of Figure 2 and agree that the sequencing was unclear and contained labeling errors. We have revised the figure to ensure the brief intervention is correctly positioned between the pre- and post-intervention OAT interest assessments. These revisions clarify the temporal flow of the intervention and assessment process. We have maintained the two interests pre- and post-intervention, respectively, because each bar represents a different group using the level of interest using the >= 8 cut-off as per the ROC.

Comment 48:
**Initial Comment:** The findings do not support this sentence: ‘...this modified SBIRT strategy deployed in probation by trained interventionists was effective at improving the uptake of MOUD.’ Revise to reflect actual findings (change in interest, not uptake).
**Authors’ Response**: We revised the sentence to more accurately reflect our results:
“This modified SBIRT strategy deployed in probation by trained interventionists was effective in identifying individuals with OUD and increasing their interest in MOUD. The intervention also resulted in a substantial increase in the number of people with OUD initiating MOUD relative to before the intervention” (Line 1088 - 1091)
**Reviewer response**: It is not clear from the Results, how the authors reach the conclusion that ‘The intervention also resulted in a substantial increase in the number of people with OUD initiating MOUD relative to before the intervention.’ What estimate are the authors comparing to? By design, none were on MOUD at baseline (i.e., an inclusion criterion was not currently on MOUD), – so are authors comparing to 0? Suggest revising Results and/or Conclusions to be clear on this point.

**Authors’ response:** We thank the reviewer for this important clarification and agree that our prior wording implied a comparison that was not supported by the study design. Because participants were not receiving MOUD at baseline by eligibility criteria, we have revised the Results and Conclusions to describe MOUD initiation descriptively rather than as an increase relative to baseline. All language implying causal improvement in uptake has been removed, and conclusions now emphasize increased interest and post-intervention initiation as outcomes warranting further controlled evaluation.

Please do not hesitate to reach out should you have any additional questions.

Best,

Matthew Ponticiello
