# Supplementary material for: Probation as a targeted entry point for scaling up opioid agonist therapies in Moldova using a modified screening, brief intervention and referral to treatment strategy
Source: PLOS Glob Public Health. 2026 May 27;6(5):e0006317. doi: 10.1371/journal.pgph.0006317 (PMC13215523; doi:10.1371/journal.pgph.0006317)
Supplement: Attachment — Submitted filename: R2R Revision 4_Final.docx [file pgph.0006317.s005.docx]

February 25^th^, 2026

Professor Kyobutungi and Pai

Editors-in-Chief, *PLOS Global Public Health*

Dear Drs. Kyobutungi and Pai,

We would like to thank both reviewers for their thoughtful review of our manuscript entitled “*Probation as a targeted entry point for scaling up opioid agonist therapies in Moldova using a modified screening, brief intervention and referral to treatment strategy”* (**PGPH-D-24-02730**). Reviewer 1 believed that all the criticisms had been addressed. For the other review, we provide point-by-point responses to each of their comments and suggestions.

**TO THE EDITORS:**

As a final editorial refinement, we re-reviewed the entire manuscript for internal consistency and clarity and noted overlapping content between the Introduction and the Methods (Context). We revised both sections to minimize repetition, focusing the Introduction on rationale and study aims while consolidating setting-specific details within the Methods (Context) section. This revision is reflected in the tracked changes.

A newly published paper on opioid agonist treatment and the legacy influence of Russian narcology in Eastern Europe and Central Asia has recently been published. In light of its relevance to our interpretation of persistent stigma and misinformation about MOUD in the region, we added one brief sentence to the Discussion to contextualize our findings. This addition does not change our results, analyses, or conclusions, but strengthens the regional framing of why brief interventions may have limited effects on attitudes and knowledge in these settings.

**Reviewer comment:** One last comment on the comparison of MOUD scores – pre and then post-intervention – using the Wilcoxon signed-rank test for paired data. In the Abstract and main paper, the authors note that ‘MOUD interest did not increase significantly after the brief intervention although post-intervention scores were shifted toward higher values overall (Wilcoxon signed-rank test, p<0.001).’ It looks that the median/IQR at both time points is 5 (3-7) but the test p-value was <0.001 meaning there is a significant difference in the median score? Is that correct? The data presented and p-value seem to conflict so suggest the authors clarify and, if needed, update inferences/conclusions, made based off of this comparison.

**Response:** Thank you for this careful observation. The reviewer is correct that the current wording is internally inconsistent and may imply a contradiction between the summary statistics and the Wilcoxon signed-rank p-value. We have revised the text for clarity. Specifically, the Wilcoxon signed-rank test evaluates within-person paired shifts in scores and does not require a change in the reported median/IQR summaries (particularly when values are rounded) to yield a statistically significant result. Thus, a significant p-value can coexist with similar pre/post medians if many participants show small upward shifts. We have revised the interpretation to reflect a statistically significant shift toward higher post-intervention interest scores, while noting the modest magnitude of change.

Revisions in manuscript text:

In Abstract/Results:
Self-reported interest in MOUD increased modestly after the brief intervention, with a statistically significant within-person shift toward higher scores (Wilcoxon signed-rank test, p<0.001).

In main Results (if medians remain the same after verification):
Although the pre- and post-intervention summary medians were similar, paired analysis showed a statistically significant upward shift in MOUD interest scores after the brief intervention (Wilcoxon signed-rank test, p<0.001), indicating small but consistent within-person increases.

Please do not hesitate to contact us if there are any remaining questions.

Best,

Matthew Ponticiello
